# Investigating the Interplay between Air Pollution and APOE Clusters in Taiwan: Implications for Alzheimer’s Disease Susceptibility

**DOI:** 10.1002/alz.086880

**Published:** 2025-01-03

**Authors:** Jong‐Ling Fuh, Yen‐Li Lo, Yung‐Shuan Lin, Wei‐Ju Lee, Cathy SJ Fann

**Affiliations:** ^1^ Taipei Veterans General Hospital, Taipei Taiwan; ^2^ National Yang Ming Chiao Tung University, Taipei Taiwan; ^3^ Academia Sinica, Taipei Taiwan; ^4^ National Yang‐Ming University Schools of Medicine, Taipei Taiwan; ^5^ Taichung Veterans General Hospital, Taichung Taiwan

## Abstract

**Background:**

There is a strong genetic component to Alzheimer’s disease (AD), as evidenced in genome‐wide association studies (GWASs) that have identified new variants associated with the disease. This is particularly true for the apolipoprotein E (APOE) gene and its neighboring genes on chromosome 19q13.3. In addition, air pollution was linked to aggravating the development of AD. A significant gap remains in the understanding of gene‐environment interactions influencing AD susceptibility. The purpose of this study is to examine how air pollution interacts with APOE clusters in Taiwan.

**Method:**

We conducted a district‐matched case‐control study involving 3,229 AD patients and 12,916 controls aged 65 to 71 years old using data from the Taiwan Precision Medicine Initiative and the Taiwan Biobank. From 2005 to 2014, air pollutants with a size up to 2.5 micrometers (PM2.5) were sampled at one‐day intervals and assigned according to home addresses according to the Taiwan Administrative Region Map. When the PM2.5 concentration exceeded 35 g/m3, it was considered a high PM2.5 day. Logistic regression models were used to assess the association of air pollution, categorized into tertiles as low, moderate, and high exposure, with the risk of AD.

**Result:**

The role of single nucleotide polymorphisms (SNPs) in air pollution has been demonstrated, especially for SNP rs147466807 (NECTIN2), when compared to PM2.5 (p = 0.005). A significant increase in AD risk was found among individuals living in high PM2.5 areas (more than 165 days exposed to high PM2.5 per year) who had one NECTIN2 risk allele compared to those without (adjusted odds ratio, 3.54; 95% confidence interval, 1.60‐7.03). Additionally, NECTIN2 6‐SNP haplotypes exhibited a similar gene‐environment interaction.

**Conclusion:**

We found a possible interaction between NECTIN gene polymorphisms and outdoor air pollution, emphasizing the importance of including environmental exposures in genetic etiological studies of AD.

